# Influence of transcutaneous electrical stimulation on heterotopic ossification: an experimental study in Wistar rats

**DOI:** 10.1590/1414-431X20153426

**Published:** 2015-08-18

**Authors:** T.G.G. Zotz, J.B. de Paula

**Affiliations:** 1 Departamento de Fisioterapia, Escola de Saùde e Biociências, Pontifícia Universidade Católica do Paraná, Curitiba, PR, Brasil; 2 Departamento de Medicina, Universidade Estadual de Ponta Grossa, Ponta Grossa, PR, Brasil

**Keywords:** Heterotopic ossification, Experimental model, Electrical stimulation, Functional electrical stimulation, Transcutaneous electrical nerve stimulation

## Abstract

Heterotopic ossification (HO) is a metaplastic biological process in which there is newly formed bone in soft tissues, resulting in joint mobility deficit and pain. Different treatment modalities have been tried to prevent HO development, but there is no consensus on a therapeutic approach. Since electrical stimulation is a widely used resource in physiotherapy practice to stimulate joint mobility, with analgesic and anti-inflammatory effects, its usefulness for HO treatment was investigated. We aimed to identify the influence of electrical stimulation on induced HO in Wistar rats. Thirty-six male rats (350-390 g) were used, and all animals were anesthetized for blood sampling before HO induction, to quantify the serum alkaline phosphatase. HO induction was performed by bone marrow implantation in both quadriceps of the animals, which were then divided into 3 groups: control (CG), transcutaneous electrical nerve stimulation (TENS) group (TG), and functional electrical stimulation (FES) group (FG) with 12 rats each. All animals were anesthetized and electrically stimulated twice per week, for 35 days from induction day. After this period, another blood sample was collected and quadriceps muscles were bilaterally removed for histological and calcium analysis and the rats were killed. Calcium levels in muscles showed significantly lower results when comparing TG and FG (P<0.001) and between TG and CG (P<0.001). Qualitative histological analyses confirmed 100% HO in FG and CG, while in TG the HO was detected in 54.5% of the animals. The effects of the muscle contractions caused by FES increased HO, while anti-inflammatory effects of TENS reduced HO.

## Introduction

Since 1883, under certain pathological conditions, bone formation has been observed in extraskeletal tissues (1). This is called heterotopic or ectopic bone formation, considered as true bone, and contains all the morphological and metabolic bone tissue, including bone marrow (BM) (2). The formation of heterotopic bone may be due to muscle trauma (myositis ossificans) (3). It is common in people who have undergone total hip arthroplasty (4,5), those with spinal cord injuries (6), and victims of head trauma (7,8), all of which often lead to long periods of immobilization of the affected limbs.

About 10% of the cases are symptomatic heterotopic ossification (HO), resulting in limitations in range of motion (6,9). Although its etiology is not fully elucidated, it has been proven that its onset is associated with damage and inflammatory processes in soft tissue (9). Based on this information, various treatment modalities have been tried to control the development of HO, but few have been effective in preventing it and not without inherent risks (3,9). Considering the treatments used to promote analgesia and consequently improve range of motion, some physical therapy resources could be useful tools for HO prevention or treatment.

In this sense, the therapeutic use of electric currents is one of several resources used in physical therapy. Once modulated with appropriate parameters, these currents can act in several different roles such as analgesia, muscle contraction, improved local circulation, fluid drainage, toning or muscle relaxation, improved regeneration, and healing of tissue (10).

Among the different forms of these electrical current applications, two were selected because they help in the kinetic functional recovery of individuals, producing analgesia postoperatively and muscle contraction to prevent atrophy due to downtime: transcutaneous electrical nerve stimulation (TENS), used as an adjunct method of analgesia in postoperative patients (11), and functional electrical stimulation (FES), used to reduce healing time and prevent muscle atrophy in the spinal cord (12).

Although it has not been elucidated whether all the injurious effects of immobilization on the tissues can be completely reversed with remobilization techniques (13), it is known that electrical nerve stimulation may be a method of prophylactic and therapeutic treatment and may contribute to the regression or prevention of HO. However, there is a need for further studies to verify those results (14).

Despite technological advances in relation to the effects of electric currents in physical therapy, the effects on HO are not known. To obtain such knowledge, it is necessary to use animal research to determine what happens in the body, with the purpose of contributing to a model that can be used in humans. Therefore, the aim of this research was to identify the influence of FES and TENS on HO in Wistar rats.

## Material and Methods

### Sample

An experimental, randomized, controlled and cross-sectional study was conducted. This research was submitted to the CEUA (Ethics Committee of Animal Use) of the Pontifícia Universidade Católica do Paraná (#499/09).

The animals were grouped into 9 standard plastic cages, each containing 4 animals, and they were numbered from one to four and maintained under controlled environmental conditions (12:12-h light-dark cycle), with free access to water and pelleted food. The study was conducted according to Federal regulation 11794/08 and the recommendations of the Brazilian College of Animal Experimentation (15).

Prior to the procedures described herein, all animals were anesthetized by intramuscular injections of 80 mg/kg ketamine and 8 mg/kg xylazine, receiving a booster dose, if necessary.

The first blood collection was from intracardiac puncture, 1 week before HO induction (16), giving the animals time to recover from the blood collection and any eventual blood loss. The experiment lasted 43 days: day 1 was the first blood collection, day 8 was HO induction, and day 43 was the day the animals were killed. The period between BM implantation and death was 35 days.

The sample consisted of 36 *Rattus norvegicus* (adult, albino, male Wistar), weighing 350-390 g. The size of the animals was essential to facilitate blood collection and placement of electrodes. The animals were randomly divided into 3 groups after HO induction as follows: control group (GC), subject only to the HO induction protocol (n=12); FES group (FG), submitted to HO induction and FES protocols (n=12); and TENS group (TG), submitted to HO induction and TENS protocols (n=12).

The animals were identified by a numeric code marked on the tail (1 through 4), and the boxes were numbered 1 through 9.

### HO induction method

All animals were anesthetized by intramuscular injections of 80 mg/kg ketamine and 8 mg/kg xylazine, receiving a booster dose, if necessary. BM was collected bilaterally from the iliac crest of the animal with a 25×17 mm puncture needle. After collection, BM was implanted bilaterally in the quadriceps. For the implant, a thin needle (0.3×16 mm) for insulin injection was inserted perpendicularly to the ventral side of the thigh. All animal groups received 0.35 mL of BM and were given oral doses of 20 mg·kg^−1^·24 h^−1^ dipyrone (500 mg/mL, Eurofarma, Brazil) during the first 3 days for pain relief after the BM collection procedure (16).

### Electrical stimulation protocol

A digital electrical stimulator (FES VIF 995, 4 channel; QUARK, Brazil) was used for quadriceps contraction. Before using the device, a test measurement (17,18) was performed, using a digital Oscilloscope (Tecktronix THS 710A, USA) in real time, which was a portable battery-powered oscilloscope to avoid interference from external sources. The unit operated at constant current (70 mA). The duration of therapy was controlled by a stopwatch. Silicon-carbon electrodes with a 1-cm diameter circular shape were used, because they presented lower impedance when compared to self-adhesive electrodes. To evaluate the impedance of the electrodes, we used a Tektronix DMM 914 multimeter, which detected 17 Ohm for the silicon-carbon and 3 kOhm for the self-adhesive electrode.

The electrodes were placed on the motor points as follows: one channel (two electrodes) was placed on the groin and one above the knee. The first was placed on the inguinal region, near the origin of the vastus medialis, and the second at a distance of 0.5 cm from the knee, with a distance of 2 cm between the electrodes. To improve the contact area of the electrodes with the skin, the animals were shaved in the hindquarters, and a gel layer was applied to protect the animal from burns and facilitate current conduction (19).

The parameters for the application of FES and TENS were defined from the calibration curves of the device in relation to the current amplitude, frequency, and duration of stimulation pulse parameters as determined for FES (Table 1) and TENS (Table 2).

**Table:**
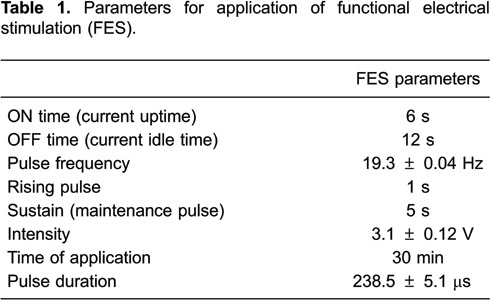

**Table:**
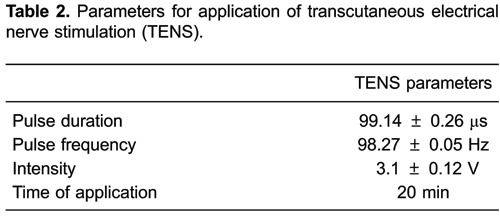

The conventional TENS was applied to the sensory level. The intensity of the sensory level was determined by increasing this parameter up to the observation of muscle contraction, when it was reduced below the level of contraction (20).

### Material collection

The rats were anesthetized to collect blood samples for the measurement of alkaline phosphatase (ALP) and for the dissection of the left and right quadriceps. During dissection, the muscles were periodically applied with an isotonic saline drip to prevent tissue drying. Under anesthesia, death was induced with a lethal intracardiac dose of anesthetic. From each group, we collected 24 muscles for analysis, 12 for histological analysis, and 12 for spectrophotometric calcium measurement. The intracardiac blood for ALP determination was collected at day 1 and at day 43 (16).

#### Histological analysis

At the end of the experiment, all collected muscles were cut across the belly, on the region where the BM was implanted. Each half was again cut into 5-mm slices, leaving the muscle divided into four pieces. These four pieces were placed in paraffin and labeled. They were then sectioned into 8-μm slices with an Olympus microtome (USA) for the preparation of histological slides. They were stained with hematoxylin and eosin and Mallory's trichrome. Each slide was microphotographed under 40, 100, and 200× magnification and used to search for implantation tissues.

#### Spectrophotometer analysis

The analysis was performed to measure the calcium levels in the left quadriceps with a Spectr AA 250 Plus (Varian, USA) flame spectrophotometer. Before analysis, the sample was subjected to a liquefaction process (21,22) in order to produce a homogeneous sample of biological material. The spectrophotometer was calibrated, using the most intense emission line of calcium at 422.7 nm.

### Statistical analysis

The results are reported as mean, median, minimum, maximum, and standard deviation. Analysis of variance with one factor was used to compare the group results in weight variables. To evaluate the effect of time on the weight (pre×8 days×43 days), we used the model of variance analysis with repeated measures. Multiple comparisons were made by the least significant difference test. In relation to the ALP variable, a comparison between pre- and post-43 days was performed using Student's *t*-test for paired samples. The normal condition of the data was evaluated using the Shapiro-Wilks test. P values less than 0.05 were considered to be statistically significant. Data were analyzed with the computer program Statistica v.8.0 (USA).

## Results

**Figure 1:**
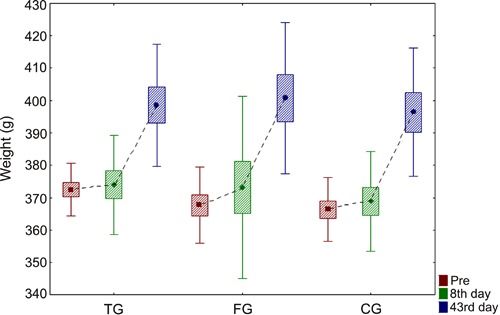
Comparison of weight within each group. Data are reported as averages, standard deviation and maximum and minimum errors. CG: control group; TG: transcutaneous electrical nerve stimulation (TENS) group; FG: functional electrical stimulation (FES) group. ANOVA was used for statistical analysis (P>0.05).

The study began with 36 animals divided into 3 groups, but 5 died before the end of the study, and 31 animals were left for final analysis. There were 2 losses in FG and CG, and one in TG. There was a weight gain within each group (Figure 1). Figure 1 illustrates the evolution of the mean weight of the animals, standard deviations (rectangles), and maximum and minimum values (vertical lines) for each group.

**Figure 2:**
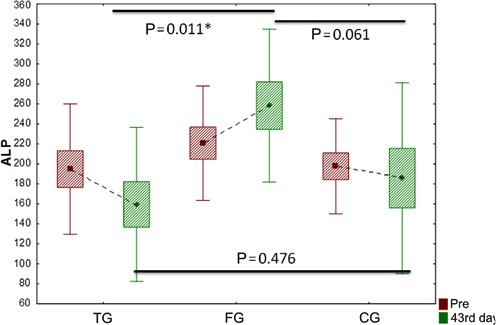
Comparison of alkaline phosphatase (ALP) among groups. Data are reported as averages, standard deviation and maximum and minimum errors. TG: transcutaneous electrical nerve stimulation (TENS) group; FG: functional electrical stimulation (FES) group; CG: control group. Student’s *t*-test was used for statistical analysis (*P<0.05).

In the evaluation of ALP on day 43, there were significant differences between the averages of the three groups. Note that ALP was significantly lower in group TG than in group FG at day 43 (P=0.011; Figure 2).

**Figure 3:**
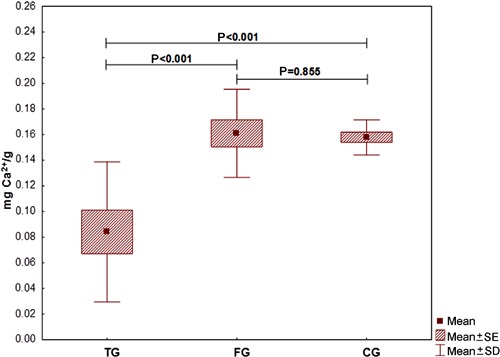
Comparison of the amount of calcium among groups. Data are reported as averages, standard deviation and maximum and minimum errors. TG: transcutaneous electrical nerve stimulation (TENS) group; FG: functional electrical stimulation (FES) group; CG: control group. The LSD test was used for statistical analysis (P<0.05).

When comparing the amount of calcium in the quadriceps muscle between groups, there were statistically significant lower results for group TG compared to groups FG and CG (P<0.001; Figure 3).

**Figure 4:**
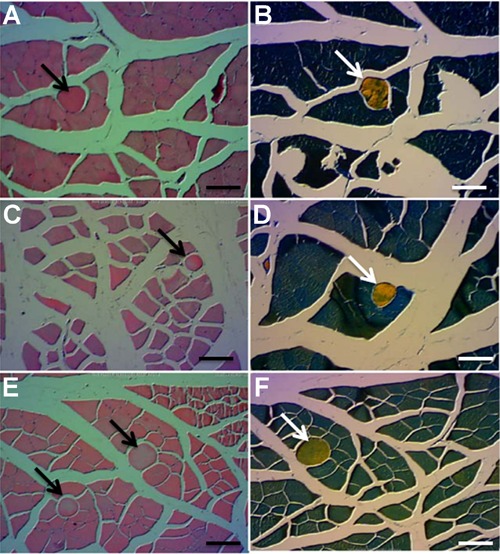
*A*, Control group, presence of heterotopic ossification (arrow) stained with hematoxylin and eosin (HE). *B*, Control group, confirmation of heterotopic bone formation (arrow) with Mallory's trichrome. *C*, Functional electrical stimulation (FES) group, presence of heterotopic ossification (arrow) stained with HE. *D*, FES group, confirmation of heterotopic bone formation (arrow) with Mallory's trichrome. *E*, Transcutaneous electrical nerve stimulation (TENS) group, presence of heterotopic ossification (arrow) stained with HE. *F*, TENS group, confirmation of heterotopic bone formation (arrow) with Mallory's trichrome. Scale bars = 100 µm.

A qualitative histological analysis was performed to evaluate the presence of heterotopic bone formation in the muscle tissues. There was HO in all animals of FG and CG, and only 7 of 11 animals showed heterotopic bone formation in TG (Figure 4).

## Discussion

It is known that HO is a condition that can lead to a decreased range of motion, and can even cause pain when there is no loss of sensitivity (6,23). The most commonly affected site is skeletal muscle (24). According to Uezumi et al. (24), skeletal muscle serves as a physical safeguard for the other organs and is anatomically located immediately beneath the skin, so it represents the most damaged organ in the body. Although skeletal muscle is characterized by the presence of fatty and connective tissues that originated from nonmyogenic mesenchymal progenitors, those progenitors were initially identified in BM (25). Therefore, HO has been thought to result from inappropriate differentiation of progenitor cells, induced by a pathological imbalance of local or systemic factors (24).

In this study, the mechanism used to experimentally induce HO formation was based on the presence of mesenchymal progenitors in BM that was collected and introduced into skeletal muscle of rats.

In addition to the concern about the mechanisms of heterotopic bone formation, the careful choice of treatment is important. Several forms of treatment are proposed to prevent or combat the development of heterotopic bone, but many have unwanted side effects (26,27).

Some physical therapy techniques can be even worse than HO (7,26). When there is no sensitivity in the patient, even passive movements can cause limb tissue shear, triggering a local inflammatory reaction, which can aggravate HO when present, or unleash it.

Therefore, this study tested the use of electrical stimulation for HO. This therapy is suitable for improving range of motion and decreasing pain (27,28). When applied with the appropriate parameters, the treatment is effective.

When the goal is to gain muscle contraction, Arantes et al. (29) reported that FES is better suited to induce action potentials in the motor nerve, causing activation of motor units. According to Rushton (30), TENS is one of the main therapeutic electrical currents used in acute and chronic pain conditions. This treatment has been used extensively in healthcare centers for the symptomatic management of acute and chronic pain of benign origin, and also in the palliative care of pain caused by metastatic bone diseases and neoplasms (31).

This research proposed to apply FES and TENS stimulation to determine their influence on induced HO in Wistar rats. A common result of that stimulation, verified in animal studies, specifically in rats, is weight loss. In this study, despite the stress of the handling and anesthesia procedures, there was a weight gain in most animals. It can be inferred that, despite the constant manipulation and anesthesia of the animals in this study, there was no significant interference to cause a loss of weight (32,33).

By comparing the behavior of the variable ALP between groups, it was observed that, at the beginning of the study, all groups were equal. On day 43, CG and TG showed mean value gains, whereas FG showed increased levels of ALP, which showed statistically significant differences when compared to FG and TG.

It is known that the examination of bone ALP is specific to assess osteoblast differentiation and mineralization increases as bone ALP values decrease, which leads to an increase in osteocalcin, a specific biochemical marker for bone formation (34). Despite the specificity of these tests, this research used ALP, because it is a tracer of osteoblast activity and also has been indicated as a diagnostic method for HO (35). However, it is known that, besides being present in bone, ALP is also present in the intestine, liver, kidneys, and placenta. Despite its presence in these organs, the increase in concentration may be related to the increase in the amount of calcium in the joints and muscles (35). The results showed a 97.5% specificity to detect bone activity (35).

The evaluation of calcium concentration in the muscles was performed by flame spectrophotometry. To this end, the muscles were weighed to calculate the amount of calcium per gram of muscle. In the case of heterotopic bone presence, the muscles would be heavier; however, this was not detected. The levels of calcium were statistically lower when comparing TG with FG or CG.

Muscle contraction occurs by the deposition of calcium in muscle tissue, and this stimulates the sliding of actin and myosin myofibrils, which characterizes the contractile process (36). In this research, this contractile process was generated artificially by electrical stimulations in the parameters of FES. It could be said that the increased levels of ALP in FG, and no significant difference in calcium concentration in TG, are related to the artificially stimulated muscle contractions in FG. This generated an increase in the number of contractions and increased local blood flow and deposition of calcium in the region of BM implantation, which may have contributed to the process of heterotopic bone formation. An explanation for the means by which FES stimulated HO formation is that electrical stimulation, when applied at the level of motor threshold in regions of bone fracture, promotes increased local vascular permeability in the BM and is related to the process of ossification (37). According to Ijiri et al. (37), electrical stimulation helps the deposition of calcium, causes changes in oxygen content and pH, stimulates expression of growth factors, and recruits help in osteoblast migration and secretion of extracellular matrix (ECM), leading to bone formation.

Mechanotransduction refers to the process by which the body converts a mechanical stimulus into a cellular response (33,36). This cellular response promotes structural changes in muscle. Physical exercise or electrical stimulation act as a mechanical stimulus (37), which promotes a physical disturbance to the cells, which is transformed into a variety of chemical signals intra- and extracellularly. The mechanical stimulus generated is transmitted to the muscle fiber through ECM, passing by the sarcolemma, and reaching intracellular molecules and the contractile system (33,36). In this sense, it is believed that different parameters of electrical stimulation can cause different results, as verified in this study.

We found a reduction in ALP and muscle calcium levels in TG. Because this form of stimulation has an anti-inflammatory action (38), the inflammatory process generated by the BM implant possibly regressed after TENS application. Moreover, the endogenous opioids released by the TENS application reduce the deposition of calcium in the muscle tissues during this stimulation (39). Gopalkrishnan and Sluka (40) compared the high frequency of TENS application (100 HZ) and low frequency (4 Hz) with different pulse durations (100 and 200 µs) for 20 min, in rats subjected to induction of the inflammatory process on their paws. They verified that the TENS of high frequency with a pulse duration of 100 µs significantly reduced the inflammatory process and pain in animals.

Thus, the process of heterotopic bone formation may have been delayed in some animals. In CG, there was a slight decrease in the ALP levels, and, comparing this group with FG, there was a statistically significant difference showing that FG presented higher levels of ALP.

The literature reports that an accommodation stimulus normally occurs, and this may undermine the expected effects of the treatments. Therefore, it is necessary to increase the current intensity or change other parameters to continue generating a sufficient stimulus (37). This did not occur in this study. Independent of stimulation, the parameters remained the same from start to finish.

For the visualization of bone formation, histological analysis was chosen, because the volumes of BM implant bone formation were small, and would be difficult to see in conventional radiography. Radiographic findings of experimental research with small volumes of implant can prove to be difficult to visualize bone formation. Some opacity, when viewed radiographically, can be inconclusive as to whether it is bone tissue resulting from edema or simply implant material that induces HO. Although the analysis of ALP and calcium levels showed a decrease in TG, bone formation was confirmed by histology in 54.5% of the animals in this group (6 of 11). FG and CG showed bone formation in 100% of the animals.

In studies using implants into the quadriceps of rabbit BM (3), new bone formation was seen in CG and the groups using anti-inflammatory agents. As far as it is known, this is the first study that verifies the influence of electrical stimulation on induced HO.

As a suggestion for future work, it would be of interest to monitor the effects of stimulation at 7, 14, 21, and 35 days, and even longer times (60, 90, and 120 days), in order to visualize the evolution of the BM implant. Another line of research that is needed would be to compare different parameters of electrical stimulation on HO, so that the indication for the use of this resource becomes safe and evidence based.

Whereas FES promotes muscle contractions, which consequently increases the local blood flow and promotes increased secretion of extracellular matrix, it was found that the influence of FES with a frequency-modulated stimulation of 19.3±0.04 Hz (pulse duration 238.5±5.1 µs, 6 s on time, 12 s off time, magnitude of stimulation 3.1±0.12 V, 30 min duration) showed enhanced effects on heterotopic bone formation.

On the other hand, the anti-inflammatory effect of TENS with a frequency-modulated stimulation of 98.27±0.05 Hz (pulse duration 99.14±0.26 µs, range of current 3.1±0.12 V, and 20 min duration) showed inhibitory effects on experimental heterotopic bone formation.

Thus, the application of calcium nerve stimulation using TENS appears to be a viable treatment option for HO.
